# Supplementary material for: Are carbon emissions trading and green financial instruments synergistic? -Comprehensive quantitative research based on content analysis
Source: PLoS One. 2024 Mar 7;19(3):e0298601. doi: 10.1371/journal.pone.0298601 (PMC10919601; doi:10.1371/journal.pone.0298601)
Supplement: S1 Data — (ZIP) [file pone.0298601.s001.zip › Data/地级市Z_F工作报告.zip!/╔╜╬≈/╜·│╟╩╨/└┤╫╘╣1⁄2╓┌║┼í╛┬φ┐╦╩2╛▌═°í┐_2012.docx]

各位代表：

现在，我代表市人民政府向大会作工作报告，请予审议，并请市政协委员和其他列席人员提出意见。

一、2011年工作回顾

过去的一年，在中共晋城市委的正确领导下，在市人大及其常委会和市政协的监督支持下，市政府团结带领全市人民，坚持以科学发展为主题，以转变经济发展方式为主线，奋力拼搏，扎实工作，圆满完成了全年各项目标任务，实现了“十二五”良好开局。全年完成生产总值895亿元，增长13.6%；规模以上工业增加值增长18%；全社会固定资产投资完成504.1亿元，增长29.5%；财政总收入完成181.8亿元，增长18.6%；公共财政预算收入完成67.9亿元，增长22.4%；外贸进出口总额完成11.4亿美元，增长113.4%；社会消费品零售总额完成224.5亿元，增长17.5%；城镇居民人均可支配收入完成20127元，增长16%；农民人均纯收入完成7043元，增长19.4%。市五届人大八次会议确定的各项目标任务和为民办的10件实事全面完成，在全省年度目标责任考核中，我市综合排名第二，荣获优秀奖。

——转型跨越思路更加明确。围绕打造全省以煤为基、多元发展的先行区和示范区，坚持以解放思想、开阔视野为先导，经过深入调研、反复论证，我们制定和完善了“十二五”规划，确立了推进转型跨越发展、再造一个新晋城的宏伟目标、战略重点和工作任务。围绕“十二五”规划的推进实施，我们立足实际，登高望远，在更大的空间谋划、设计、发展晋城，作出了一系列战略决策：提出了“对接上海，融入中原”的扩大对外开放战略，走出去承接发达地区产业转移，与大企业、大集团携手合作的氛围日渐浓厚，招商引资成为全社会的热点、焦点、兴奋点，“四个一千亿”的招商成果提振了我们转型跨越的信心；提出了要发挥晋城地处山西综改试验区、中原经济区和中部崛起三大国家战略的交汇点，辐射周边两小时半径内13个城市6000万人口的区位优势，建设区域性经济中心；提出了加快推进工业化、城镇化和农业现代化的实质是统筹城乡、统筹经济社会发展，在工作安排上进一步理清了三者之间的内在关系；按照全市一盘棋的思路，根据各地现有产业基础，进一步明确了产业发展方向和实现路径，科学确定了各县（市、区）非煤主导产业功能定位，提出了“十二五”期间用非煤产业再造一个新晋城的目标；确定了建设“方便、温馨、开放、现代”的宜居之城、活力之城、幸福之城的城市总体格调和定位。特别是通过对我市“十二五”面临的挑战和机遇分析，全市上下的认识更加统一，步调更加一致，紧迫感更加增强，转型跨越的信心更加坚定。

——结构调整步伐更加坚实。一批重大项目开工建设。启动实施了169项重点工程和重大项目，当年完成投资466.3亿元，占年度目标的133.8%，增长48.6%。资源类产业巩固提升。煤矿整合重组基本完成，煤炭产业素质发生了脱胎换骨的变化。煤层气产业，本着“企业得利，地方得税，互利共赢”的原则，与中石油华北油田公司合作，共同推动煤层气开发利用的规模化、产业化发展。国内规模最大、日处理90万立方的天煜新能源煤层气液化项目进展顺利。电力产业，国投热电一期2×30万千瓦机组开始试运行，晋煤能源公司煤矸石发电和中电明秀瓦斯发电一期工程进展顺利，高平煤电化一体化、华能阳城电厂等项目完成前期工作。煤化工产业，晋煤集团百万吨煤制油项目奠基，金象公司年产18万吨合成氨30万吨尿素项目联动试车。非资源类产业快速成长。全年非煤工业增加值增长22%，高出规模以上工业增加值增速4个百分点，全市新型工业化水平达到47.6%，居全省第二位。高新技术产业快速崛起。投资60亿元的富士康A区精密刀具及工业机器人项目4月开工建设，9月就正式投产。投资30亿元的B区继续提升产能。富士康金匠科技工业园新区项目开工建设。未来5至10年，富士康将投资1000亿元实施金匠中原计划，在晋城打造全球精密制造之都。以精密铸造、煤机成套设备为主的装备制造业规模扩大，增加值增长57.8%。金鼎煤机金匠园区开工奠基，福川制铁项目加快推进。清慧汽车配件及大型专用铸造项目二期工程试生产。纽威信10万吨精密数控机床项目在高平成功落地。商贸物流业发展壮大，豪德贸易广场一期交付使用，二期奠基开工。国贸大厦竣工开业，一批国际国内知名的商贸企业落户晋城。白马寺森林公园太行明珠游乐城项目开工建设，苏宁电器确定了选址，上海新世界、红星美凯龙、丹尼斯、上海锦江酒店等一大批项目正在深度对接。文化旅游业亮点频现，举办了第四届中国（晋城）太行山国际旅游文化月等旅游推介活动。以泽州府城玉皇庙的二十八宿雕塑为原型，与央视动画公司联合制作的大型原创动漫片《大耳朵爷爷历险记》首部52集，于今年春节期间在央视少儿频道热播。成功竞标央视一套黄金时段广告，从2012年起播放晋城的城市形象推介宣传片。

——“三农”工作成效更加突出。建立倒推倒逼机制，对农民增收的四个要素层层分解、条块结合、分工负责、逐项落实，农民收入增幅创近年新高。粮食总产量达9.2亿公斤。农业综合机械化水平达到61.1%，高出全国、全省6.6和5.9个百分点。畜牧业生产稳定增长，全年生猪存、出栏分别增长8.5%和11.1%。农业产业化发展迅速，新发展农民专业合作社427个，农产品加工转化率达到41%。大力推进水利改革发展，市水务发展有限公司挂牌成立。积极创建国家森林城市，完成造林22.78万亩，森林覆盖率达到39.2%，居全省首位。强力推进扶贫攻坚，启动了第三批223个扶贫攻坚重点帮扶村的推进工作。扎实开展新农村建设，新的农村“五个全覆盖”工程中，新型农村社会养老保险、中等职业教育免费和农村文化体育场所三个全覆盖已提前一年完成。

——城市基础设施更加完善。积极推进城市形象设计，搞好城市建设用地科学规划、合理利用，统筹推进城市基础设施建设。启动实施了5条城市道路工程建设，完成投资近5亿元，其中凤城南路建成通车。兰花路、红星西街、红星东街开工建设，泽州路、凤台街罩面改造工程全部完成。困扰老城区居民多年的40条背街小巷改造工程竣工，这些地区的居民再也不用担心雨天出行的泥泞。新购置的40辆公交车上线运行。总长35公里的热电联供热网工程主干管网投入使用，新增集中供热能力1227万平方米。豪德贸易广场、金村新区的供水、供热、供气等基础设施配套项目已全部启动实施。市区和各县城全部开通煤层气，主城区气化率达到90%。14416套保障性住房开工建设。城市水系建设积极推进，任庄水库调水工程白马寺温馨湖竣工，主城区花园头河景观水系、滨河南路建设工程开工奠基。城镇扩容提质大行动和百镇建设工程全面启动。沁河高标准综合治理工程正在设计。经过一年的冲刺攻坚，我市荣膺“国家卫生城市”称号。

——城乡生态环境更加改善。继续深入实施“蓝天碧水工程”，环境质量进一步提高。9家煤化工企业生产工艺废水超低排放技改工程全部完成。全市地表水环境质量持续好转，丹河、沁河出境断面水质均达功能类别要求。六县（市、区）环境空气质量稳定达到国家二级标准。市区二级以上天数达到354天，其中一级天数达到112天。强力推进污染减排，对列入省政府环境污染末位淘汰范围的12家企业或设施实施了关停、淘汰和取缔。重点实施的35个污染减排项目全部完成。白马寺山森林公园绿化上档升级，丹河两岸绿化工程稳步推进，环城、阳翼和高陵三条高速的通道绿化基本完成。继续开展生态示范创建活动，完成了4个国家级和16个省级生态村镇的创建工作。我市荣膺全球环境保护最高奖——联合国环境规划署授予的“中国区环境规划优秀示范城市”称号。

——改革开放氛围更加浓厚。研究制定了转型综改试验行动方案，加快推进“一市两县”、“一市两园”、“一县一企”试点。国企改革深入推进，民营经济蓬勃发展，医药、卫生、投融资体制改革步伐加快，文化体制改革走在全省、全国前列。进一步扩大对外开放，全力开展招商引资。积极创新招商模式，通过小分队出击、大队伍跟进、板块式招商的方法，对准上海、对准富士康、对准华润、对准晋煤开展密集化招商引资，取得了阶段性重要成果。建立了全面对接上海的顺畅通道，与上海方面签订战略框架合作协议16个，签署项目合作意向7个，达成项目口头合作意向26个。太行明珠游乐城、上海枫彩集团沁水彩色苗木花卉项目、香港东裕包装纸袋等项目奠基开工。加大与大企业大集团的战略合作，成功与华润签署了1180亿元战略合作框架协议，兰花？华润药业开工建设。加强企地合作，将晋煤集团纳入全市转型跨越的大格局中，1237亿元的投资计划正在逐项落实。第六届中博会我市共签约项目68个，拟引资2439亿元，居全省第三位。融入中原战略深入实施，与中原地区的经贸往来日益紧密。加强对目标任务的考核，“请给我结果”的方法，压出了状态，激发了士气。招商引资当年签约、当年到位资金是上年的4倍多。

——人民生活质量更加提升。科技、教育、人口计生、卫生、文化、体育等各项社会事业全面进步。继续加大对民生、重点社会事业的支出，用于教育、科技、医疗卫生、农林水事务支出的增幅均高于一般预算支出增幅。连续六届荣膺全国科技进步先进市。市中等专业学校的搬迁扩建工程、凤城中学、秀水苑学校、汇仟小学等四所学校全部开工奠基。完成了中小学校舍安全工程扫尾工作，为100所农村中小学校配备了体艺器材，为100所农村寄宿制学校增添了卫生安全设施。新建和改扩建幼儿园15所。按照又好又快又省的思路，与晋煤集团联手，加快了晋城大医院建设的节奏。与北京阜外医院合作组建了心血管病技术培训中心。公共卫生和医疗服务体系更加完善，医疗服务水平进一步提高。大力创建国家级创业型城市，新增城镇就业岗位3.37万个，城镇登记失业率控制在2.2%以内。进一步完善社会保障体系，提高了城镇职工和城镇居民的医疗保险最高支付限额，实现了城乡居民养老保险全覆盖，新农保参保率和城乡低保覆盖率居全省前列。全面完成低收入农户冬季取暖用煤工作，全市供煤总量68.3万吨，供煤标准、质量均高于往年。民族宗教、妇女儿童、统计、档案、史志、外事、侨务、文物保护、人防、气象、地震、老龄、残疾人、红十字、慈善救助等社会事业都取得了新进展和新成绩。

——和谐稳定局面更加巩固。深入推进精神文明和民主法制建设。自觉接受人大、政协监督，认真听取各民主党派、工商联和无党派人士意见。全年共办理人大代表建议意见436 件、政协提案515件。坚持依法行政，法治政府建设成效明显，“六五”普法扎实开展，荣获全国法制宣传教育先进城市。加强政风行风建设，被省政府表彰为“政风行风评议先进市”。创新社会管理机制，深入开展“严打”整治斗争，强化信访和社会矛盾排查调处工作，“平安晋城”建设成效显著，连续15年被评为全省社会治安综合治理先进市。大力深化全民国防教育，积极探索晋城特色的军民融合式发展路子。深入开展安全生产专项治理，加大隐患排查治理力度，发生事故起数和死亡人数均低于省控指标，安全形势总体平稳。

各位代表，“十二五”起步之年，我们既理清了思路，确定了路径，也收获了硕果，赢得了开局。这是贯彻执行党中央、国务院和省委、省政府一系列方针政策的结果，是中共晋城市委统揽全局、正确领导的结果，是市人大、市政协大力支持、有效监督的结果，是全市人民辛勤工作、共同努力的结果。在这里，我代表市人民政府，向全市人民，向驻市解放军指战员、武警官兵和公安干警，向各民主党派、工商联、各人民团体和社会各界人士，向所有关心和支持晋城发展的同志们、朋友们，表示衷心的感谢和崇高的敬意！

在肯定成绩的同时，我们也清醒地认识到：产业结构单一的局面没有根本改观，支柱产业多元化格局远未形成；支撑发展的大项目、好项目不多，项目推进不力的问题仍然存在；加快发展与环境容量不足的矛盾日益突出，节能减排压力较大；保持城乡居民收入持续较快增长的难度增加，改善民生、创新社会管理和加强安全生产监管的任务仍很艰巨；特别是面对全国、全省各地抢抓机遇的热情、争先发展的势头，我们一些干部的状态还没有调整到最佳、作风还不能完全适应工作要求等。对此，我们必须采取更加有力的措施认真加以解决。

二、2012年工作安排

2012年是实施“十二五”规划承上启下的重要一年，也是进一步推动转型跨越发展的关键之年。做好今年的政府工作，具有十分重要的意义。

今年政府工作的总体要求是：突出转变经济发展方式主线和稳中求进、好中求快总基调，扭住“项目落地年”这个重点，加快以煤为基、多元发展步伐，着力保持先发优势，着力创优人居环境，着力扩大开放引进，着力保障和改善民生，着力创新社会管理，在“四化统筹”中建设转型综改试验先行区示范区，坚定不移争当全省转型跨越排头兵，坚定不移加快全面建设小康社会步伐，以优异成绩迎接党的十八大胜利召开。

全市经济社会发展的主要预期目标是：地区生产总值增长15%；财政总收入增长16%，公共财政预算收入增长16%；规模以上工业增加值增长18%以上，社会消费品零售总额增长17%；全社会固定资产投资增长30%；城镇居民人均可支配收入增长13%；农民人均纯收入增长15%以上；居民消费价格总水平上涨幅度控制在4%左右。

约束性指标是：万元生产总值综合能耗下降3.5%；完成省政府下达的化学需氧量、氨氮、二氧化硫、氮氧化物、烟尘、粉尘年度减排指标任务。城镇登记失业率控制在4.2%以内。

做好今年政府工作，必须在推进转型综改试验中，寻找晋城独特的机遇，大胆先行先试，以推进产业转型为核心，带动生态修复、城乡统筹和民生改善，使发展的活力更加增强；必须以扩大开放为突破口，以招商引资为着力点，加快已签定协议和签约项目的落实，继续策划、引进、储备一批大项目、好项目，使转型的支撑更加有力；必须充分挖掘区位优势的经济内涵，拓宽视野，在更大的空间中优化配置资源，努力把我市建设成为晋冀豫三省接壤地带的区域性经济中心、金融中心、商贸物流中心、生态旅游文化和休闲娱乐健康度假中心，使跨越的势能加快集聚；必须统筹全市产业布局，围绕非煤主导产业功能定位，各县（市、区）要坚持优势互动，错位突破，集群发展，使发展的潜能充分释放；必须科学把握工业新型化、农业现代化、市域城镇化、城乡生态化的内在关系，创新机制，互促互动，以工业集聚带动人口集聚，以园区建设带动社区建设，以城镇化水平的提升，促进农业现代化、城乡生态化水平的提升，使“四化”的推进速度和质量更快更好；必须把改善民生作为经济社会发展的最高目标，把更多的人力财力物力，用到解决群众关心、社会关注的问题上，使城乡居民得到的实惠程度、对政府工作的满意程度与经济社会的发展程度同步提高。

今年，要重点抓好以下八方面工作：

（一）加快经济结构战略性调整，在培育壮大非资源产业上取得新进展。

加快推进资源类产业新型化。煤炭产业，要充分认识以煤为基的重要性，把煤炭产业作为推进转型跨越发展的重要保障和基础支撑，继续推进煤炭工业新型化。要巩固提升煤矿兼并重组煤炭资源整合成果，加快重组整合矿井复工复产和改造建设进度，今年要新增投资58亿元，竣工验收投产或联合试运转矿井14座。进一步规范煤炭行业生产、经营、建设、安全秩序，大力推广先进技术，提升从业人员素质，培育现代煤炭集团，提升规模化、机械化、现代化、多元化发展水平，提高煤炭工业经济增长的质量和效益。生产矿井采煤机械化程度达到100%，掘进机械化程度达到82%，煤炭资源采区回采率达到70%。煤层气产业，进一步理顺煤层气开发利用机制，加大技术攻关和引进推广力度。加快落实国家煤层气产业发展规划，加强与中石油华北油田的战略合作，实现互利共赢。积极推进华凯煤层气公司日产15万方、南京中油恒燃日产50万方煤层气液化项目。全年地面煤层气抽采能力达到40亿立方米，煤层气日液化能力突破200万立方米，市区煤层气气化率达到92%，煤矿瓦斯抽采利用率达到38%。煤化工产业，要按照“稳定提升基础煤化工，重点发展新型煤化工，积极发展精细煤化工，开拓发展煤层气化工，探索发展低碳型煤化工”的思路，加快淘汰落后化肥产能，整合重组上下游产业链。抓好山西兰花煤化工二期及化肥分公司的搬迁改造。电力产业，要加快构建以清洁高效燃煤发电为主体，以煤层气、煤矸石等多元发电为支撑，与智能电网建设相协调的电力产业体系。阳城晋煤能源2×13.5万千瓦煤矸石发电项目、山西中电明秀12万千瓦瓦斯发电项目建成投产。

壮大非资源类优势产业。高新技术产业，要以富士康为重点，充分发挥其在经济发展中的带动、集聚、引领作用，加快壮大高新技术产业的规模和实力，形成扩张和倍增效应。重点抓好富士康金匠科技工业园、晋煤乐百利特大功率LED芯片及节能灯具、中道能源锂离子电池、唐一锂离子动力电池、硕阳光电太阳能单晶硅项目建设。要打破常规，全力以赴，集中力量打好富士康项目落地大会战，确保项目按照时间节点顺利推进。装备制造业，围绕淘汰落后和整合提升，加快集约化发展，努力形成铸造产业集群。加快推进金鼎煤机、纽威信精密数控机床、路宝铝轮毂二期等项目，开工建设兴达公司拖拉机变速箱改扩建、清慧公司镁基合金挤压铸造等项目。现代服务业，要学习借鉴上海发展现代服务业的经验和做法，密切与上海合作，加快金村现代服务业新区建设。商贸物流业，要充分发挥地理、区位、交通和环境优势，面向中原城市群，融入中原经济区，全力打造区域性商贸物流中心。加强与浙江义乌小商品城的合作，加快推进大型物流园区和商贸市场建设，抓好豪德二期、沃尔玛等项目的落地工作。文化旅游业，编制《晋城与中原区域旅游战略合作规划》，深度融入中原市场。加大重点景区开发提升力度，着力推动陵川休闲旅游健康度假目的地建设。完善旅游配套设施，打造精品旅游线路，开发特色旅游产品，促进旅游与文化的深度融合。要利用我市在央视黄金时段城市形象广告的推介效应，大力拓展旅游市场，使我市旅游业有较大发展。

着力推进园区建设。进一步理顺园区管理体制，提升管理水平，提高服务质量，营造良好的外部环境。坚持“政府引导，市场运作，多元投入，要素整合”的原则，高起点规划建设园区的水、电、路、气、暖、网等各项基础设施建设。积极拓宽融资渠道，吸引各类主体参与园区基础设施建设。要继续抓好“一区六园”建设，按照园区的功能定位，积极引进和培育龙头企业，延伸产业链条，带动与之协作配套的中小企业集群化发展。

做大做强本土企业。把晋煤集团等中央、省属驻市企业纳入到全市发展大格局中，提供优质服务，支持多元发展，充分发挥他们在产业转型和城市提升中的重要作用。支持兰花等地方国有大型企业，整合优势资源，做大做优做强。加强国有企业班子建设，建立国企业绩评价制度，强化国有资产监管。加大政策扶持力度，大力实施中小企业成长工程。进一步放宽市场准入，拓宽民间资本投资的领域和范围，鼓励和引导民间资本进入基础产业、基础设施、社会事业、金融服务业、文化产业等领域，打造一批营业收入过10亿、50亿、100亿的“旗舰”企业。大力推进公共服务平台建设，要在“入口”上解决融资、用地等生产要素问题，在“出口”上解决财税政策的落实问题，在“中间环节”上解决技术、人才等问题。

各位代表，结构调整永无止境，转变方式任重道远。只要我们咬定目标，持之以恒，一抓到底，我市的产业结构就一定能够发生根本性变化，用非煤产业再造一个新晋城的目标就一定能够实现！

（二）夯实农业农村发展基础，在农业增效、农民增收上取得新进展。

保持农民增收的好势头。要继续实行倒推倒逼、分工负责的办法，全面提高农民工资性收入、经营性收入、转移性收入和财产性收入，确保农民收入大幅增长。要把协同推进工业新型化、农业现代化、市域城镇化、城乡生态化与农民增收结合起来，作为解决“三农问题”的新思维，成为统筹城乡发展的新机制。要建立完善以煤补农体制机制，动员资源型企业以多种方式支持“三农”。要加大农村劳动力转移就业和技能培训力度，年培训农民不少于10万人。

做大做强特色现代农业优势产业。毫不放松抓好粮食生产，以100万亩粮食丰收工程为载体，主攻粮食单位面积产量，力争用1/3的粮田拿回1/2的产量。要按照工业化的理念、产业化的运作，进一步做大做强畜牧、蚕桑、蔬菜、干鲜果、小杂粮、中药材、食用菌等特色现代农业优势产业。建设150个“一村一品”专业村，加快实现户有专业、村有规模、乡有特色、区有龙头的产业新格局。培育壮大农产品加工“513”龙头企业，延长产业链，突出抓好高平雨润、沁水大象、陵川五色土等10个亿元龙头企业。要注重农业科技创新，完善农技推广体系，通过新品种、新技术示范推广，激发现代农业发展的内生动力。

加强农业基础设施建设。按照“十二五”水利发展规划，加快“井”字型骨干大水网、城乡一体化饮水安全网、农田水利灌溉网三个大水网建设。全面完成任庄水库调水工程，开工建设沁河综合治理工程，积极推进郭壁供水改扩建、杜河提水、大磨河水库等水源工程，恢复续建曹河、磨滩水电站。继续实施“一矿一池一园区”战略，充分利用煤矿废水，建设节水灌溉工程。实行最严格的耕地保护制度，合理利用每寸土地。新增农业机械化保护性耕作面积6万亩，节水灌溉面积达到38万亩，完成中低产田改造10万亩。

扎实推进扶贫开发和新农村建设。加大政策扶持和社会帮扶力度。从今年起，每年重点扶持150个贫困村产业发展，到“十二五”末，基本消除人均收入在5000元以下的发展滞后村。以集镇为主要搬迁地，加大贫困地区易地移民搬迁力度。完善扶贫开发帮扶机制，抓好扶贫开发和农村低保制度的有效衔接。新农村建设要重点抓好北石店、巴公—高都、原村—马村等10个城乡一体化发展示范片区和300个新农村重点推进村建设。高质量推进农村街巷硬化、农村便民连锁店全覆盖，确保农村新的“五个全覆盖”工程全面完成。

各位代表，今后虽然农业份额会下降，农村人口会减少，但农业的基础地位不会改变，“三农”问题的重要性不会改变。我们要怀着对农民的深厚感情，统筹协调好各方面的关系，更加科学有效地推动农业农村发展。

（三）统筹城乡和经济社会发展，在市域城镇化上取得新进展。

进一步强化城市设计。把城市建设与产业发展结合起来，把城中村、棚户区改造、保障房建设与提升城市品质结合起来，把规范房地产开发秩序与充分提升城市土地资源利用效率结合起来，把建成区的升级改造与塑造“方便、温馨、开放、现代”的城市格调结合起来，从长远和战略的高度，在更高层次、更大空间内对城市进行创意策划。统筹推进城市规划、土地利用、产业布局、基础设施建设和生态保护，实现优势互补、经济互动、资源共享。

加大中心城市建设力度。继续加强城市基础设施建设，拓展城市空间、提升城市品位。围绕拉大城市框架，打通断头路，畅通主干道，缓解市内交通压力。实施凤台街人行道改造提升工程。打通文昌街西段，使主城区再增加一条东西大通道。新建金村大道、滨河南路和客运东站环路。兰花路、红星西街、红星东街竣工通车。新增70台公交车辆，方便市民出行。加快实施城市供水、污水深度处理、再生水利用管网配套工程。加强水系建设，加快推进主城区花园头河景观水系改造，使之成为城市片区改造、功能提升、产业发展、景观打造的一项系统工程。大幅度增加水域面积，打造林水相依、人水和谐的高标准城市水系。着力提升城市管理水平，使市容更整洁、交通更顺畅、出行更安全、景观更怡人。

加快市域城镇化进程。要按照工业园区化、园区社区化、社区城镇化的思路，以产业发展为重点，增强城镇和工业园区的辐射力、带动力。要在更高的层次统筹县域经济发展，积极推进城镇组群发展，形成分工合理、联系紧密的城镇群、经济圈和旅游带。重点推进沁河流域、丹河流域、环城高速周边等片区的特色城镇化建设。按照全省“百镇建设工程”的要求，加快北留、嘉峰2个省级重点示范镇和14个市级重点镇建设。抓好郑庄镇“三化协同推进”示范试点工作。今年全市城镇化水平要提高3个百分点以上。

创新城乡一体化发展的体制机制。加快户籍制度改革，鼓励非农产业和农村人口向城镇转移集中。进一步放宽落户条件，对在城镇有合法稳定住所和稳定职业人员，在自愿的基础上转为城镇户口，其在农村原有宅基地使用权、土地承包经营权和集体收益分配权不变。今后就业、义务教育、社会保障等政策不再与户口性质挂钩。对城市现有人口实行统一管理，推行流动人口“居住证”制度，逐步消除城乡户籍待遇差距，实现基本公共服务均等化。

（四）加强环境治理和节能减排，在城乡生态化上取得新进展。

狠抓节能降耗工作。严把高耗能项目准入关和投运关，有效控制高耗能行业过快增长。继续加速淘汰落后产能，对已列入淘汰计划的企业和生产工艺，挂牌督办，限期关停。注重结构节能，加快推进低耗能、低排放、高附加值的新兴产业和服务业的发展。全面推进工业、交通、建筑和公共机构节能，继续抓好重点行业、重点企业节能工作，完成70万平方米建筑节能改造任务。

加大环保整治力度。继续开展区域环境综合整治，保障空气质量持续改善和提升。加快建设丹河人工湿地三期工程。强力推进污染减排，建立完善排污权交易机制体制，提高项目准入门槛，切实抓好污染源治理。落实“以奖促治”和“以奖代补”政策，以实施农村环境连片整治示范项目为抓手，全面加强农村环境保护工作。

加强生态林业建设。加快国家森林城市创建步伐，持续推进造林绿化工程，完成20万亩造林任务，提升10个城郊森林公园，建设10个名优干果经济林乡镇，新建1万亩苗木基地。完成新植物园建设工程。启动申报国家生态园林城市工作。从今年起，按照“五环”、“五片”、“20条线”、70个乡镇所在地的总体规划布局，高标准推动村庄绿化工程，今年先期完成300个村庄。要因地制宜，大面积营造村庄林带、林片，让晋城的村庄恢复乡情野趣，保持田园风光。

大力发展循环经济。继续推进煤层气、煤矸石、粉煤灰、工业炉渣、农村沼气、秸秆气化为重点的资源综合利用工作。以“减量化、再利用、资源化”为目标，鼓励开展技术创新和先进适用技术的推广应用，着力发展资源节约和综合利用型产业，最大限度提高资源能源利用率。

各位代表，生态与环境是晋城人民坚持科学发展的一个最自豪的品牌。我们要无比珍惜，以对未来负责的态度，采取一切措施，让我们的后代拥有比我们更湛蓝的天，更清澈的水，更新鲜的空气，更繁茂的森林！

（五）加大创新改革与扩大开放力度，在综改试验和项目建设上取得新进展。

要善于在创新中深化改革。如果说，我们过去更多的是通过制度的改革实现发展的创新，那么，当改革进行到今天时，我们要更加善于通过发展目标的科学定位与创新，撬动制度的改革，在发展的创新中深化制度的改革。

要更加增强开放的自觉自信。在全社会营造更加浓厚的开放氛围，真正让开放成为我们的思维习惯和工作方法。要充分认识到，没有开放就没有我们的转型，没有开放就没有我们的跨越，没有开放就不能拓展我们的未来，没有开放就不能保持我们发展的优势。招商引资是开放的核心。要深入实施“对接上海，融入中原”战略，通过几年甚至是十几年坚持不懈的努力，把上海耕耘成晋城招商引资的一块熟地，把晋城变成上海产业转移的一块飞地。要积极融入中原，利用资源产业优势，增强辐射功能。以主导产业为纽带，和周边城市的产业形成产业链条关系；加深商贸物流融合，积极拓展市场空间，力争建设成为晋豫接壤区域人流、物流、信息流汇集的购物首选地；加强旅游合作，增强旅游休闲产业的覆盖面，共同打造“太行山文化旅游”精品，把晋城打造成晋东南及中原经济区的生态旅游文化中心和休闲娱乐度假中心。各县（市、区）要按照各自的非煤主导产业定位建设园区，通过招商引资，引进产业链条上的中小企业集聚。要完善目标责任制，让每个领导干部头上都有招商引资指标，每个部门都有招商引资任务。要积极主动走出去，通过政府高层对接、部门对接、中介组织对接、企业对接，把去年与上海、富士康、华润、晋煤签订的协议化为一个一个的落地项目，化为一年一年的投资计划。在此基础上要争取再谋划签约一批大项目、好项目，使招商引资工作继续走在全省前列。

要深入开展“项目落地年”活动。把招商引资和项目建设作为各级领导干部的重要责任、重要能力、重要政绩。建立招商项目跟踪落实工作机制，提高签约项目落地率。重点抓好“四个千亿”项目对接、233项重点工程和100项工业转型项目建设。要把项目落实到责任部门，实行主要领导第一责任人制度，做到责任主体、职责任务、工作标准、时限要求“四明确”。实行定期汇报、监督检查、跟踪问效的工作机制，建立项目落地和项目建设月报表、季检查、半年通报、年终考评制度，力促项目落实。建立重点项目、工作亮点观摩制度，每半年不仅要对各县（市、区）的重点项目进行观摩，而且要对各部门的工作亮点进行观摩。

要全面推进转型综改试验区建设。要抓住产业转型这个核心、企业发展这个主体、园区建设这个载体、项目落地这个关键，以产业转型带动生态修复、城乡统筹、民生改善。要加快推进“一市两县”、“一市两园”、“一县一企”先行试点建设，确定一批影响大、带动性强、示范意义明显的标杆项目、标杆工程、标杆园区、标杆企业、标杆企业家。要突破体制机制这个难点，在增强转型推动能力，“四化”联动机制，建设“区域经济中心”，缓解“项目瓶颈”，以煤补农、以煤补绿、以煤促文，理顺煤层气开发体制等方面先行先试，确保快速启动、高点起步，力争在“综改区”建设上走在全省前头。

（六）提升公共服务水平，在保障和改善民生上取得新进展。

进一步扩大社会就业。把充分就业作为经济社会发展的优先目标，深入推进国家级创业型城市创建活动，确保全市城镇新增就业2.38万人、下岗失业人员再就业5500人、就业困难人员就业1400人。围绕我市主导产业和招商落地项目，大规模开展职业技能培训，完成城镇失业人员再就业培训5000人，新成长劳动力培训2000人。

完善社会保障体系。继续扩大社会保险覆盖面，稳步提高社会保险统筹层次和标准。加强城镇居民社会养老保险和新农保工作制度化、规范化管理，提高服务水平。巩固城镇医疗保险和新农合参保率，继续提高企业退休人员基本养老金标准。认真研究民政社会福利服务工作，以社区服务、社会救助、养老服务、慈善事业基层建设等为切入点，进行体制创新和制度设计，建立普惠型社会福利和公共服务体系。把保障性住房与园区建设相结合，有效吸收农民进城，推进城镇化。今年新开工建设各类保障性住房1.4万套。要创新资金保障机制，优先满足保障性住房建设需要，让更多的低收入群众住上价廉舒适的新房。继续严格执行房地产调控政策，促进房地产市场健康发展。

坚持优先发展教育。进一步加大教育投入，确保教育支出占公共财政预算支出比例达到19.35％。实施好农村义务教育薄弱学校改造计划，加快推进全市义务教育均衡发展。大力发展职业教育，认真落实国家对中等职业教育学生的各项资助政策。加快晋城本科院校、市中等专业学校、凤城中学、秀水苑学校、汇仟小学建设工程。加强教师队伍建设，做好教师补充和培训工作，尤其要提升师德、师风、师能。全社会都要厚待教师，创造条件使有能力的优秀教师脱颖而出，营造环境吸引优秀师资力量到晋城任教。同时，对品德、作风、能力都不足以为人师表的人，要坚决调离教师队伍。继续抓好学前教育、民办教育、继续教育和特殊教育，促进各级各类教育全面协调发展。

大力发展科技、卫生、文化、体育等社会事业。继续把推进科技创新放在重要位置，建立以企业为主体的技术创新体系，推进高技能人才队伍建设，让敢为人先、勇立潮头的创新创业人才，拥有更大发展空间、赢得更多信任宽容、获得更好扶持帮助。健全公共卫生服务网络，推进医疗卫生机构之间的资源共享、协调互动。加强食品安全综合协调和安全监管，积极预防重大传染病发生，提高重大突发公共卫生事件处置能力。加快晋城大医院、市儿童医院扩建和市二院住院部大楼建设，有效提升全市医疗服务水平。切实加强人口和计划生育工作，促进人口长期均衡发展。继续深化文化体制改革，推动文化大发展大繁荣，加快公共文化基础设施建设，增强公共文化服务能力。继续推进文化低保工程，推进文艺精品创作生产。扶持公益性文化事业，大力发展文化产业，满足人民群众日益增长的精神文化需求。开工建设晋城大剧院，完成市美术馆、图书馆、档案馆“三馆合一”主体工程。大力开展全民健身活动，促进群众体育和竞技体育协调发展。

各位代表，不断提高人民群众的生活水平是我们一切工作的出发点和落脚点，为人民群众谋福祉是我们的不懈追求。新的一年，我们要投入更多的人力物力财力，把各项社会事业发展好，把事关群众切身利益的事情办好，让人民群众生活得更富裕、更幸福、更有尊严！

（七）积极推进民主法制和精神文明建设，在维护和谐稳定上取得新进展。

加强民主法制建设。认真执行人大及其常委会的决议、决定，依法向人大及其常委会报告工作，自觉接受监督，积极支持人民政协履行政治协商、民主监督、参政议政职能，认真办理人大代表建议和政协提案。支持各民主党派、工商联、无党派人士和工会、共青团、妇联等人民团体开展工作。完善基层民主管理制度，提升基层自治组织的管理能力和水平。深入推进政务公开、村务公开、厂务公开，保障人民群众的知情权、参与权、表达权、监督权。充实完善“六级六步”大调解内容，健全和规范大调解工作制度。大力推进“阳光司法”、“阳光执法”，努力促进司法和谐，提升执法公信力。深入实施“六五”普法规划，扎实开展“法制晋城”建设。

加强精神文明建设。深入推进文明城市创建工作，巩固发展全民参与、共建共享的创建格局，使我们这座城市拥有文化认同感、情感归属感、心灵愉悦感。进一步加强社会主义核心价值体系建设，加强社会公德、职业道德、家庭美德、个人品德的宣传教育，大力弘扬晋城精神，人人争做“晋城好人”，引深公民道德实践活动。积极开展双拥工作，巩固发展军政军民团结的好局面。

加强和创新社会管理。坚持以人为本、服务为先的理念，进一步完善“党委领导、政府负责、社会协同、公众参与”的社会管理格局。深入推进社会管理综合治理和平安建设。加强基层民主和社区建设，推进基层民主自治制度化、规范化、程序化。建立社会矛盾综合协调化解的长效机制，及时妥善处理各类矛盾和敏感事件。要完善社会治安防控体系，严厉打击各种破坏和谐稳定的村霸、市霸、路霸等社会黑恶势力和严重侵害群众生命财产安全的各类违法犯罪活动。大力加强食品药品安全整治，保障人民群众饮食用药安全。大力推进政务诚信、商务诚信、社会诚信和司法公信建设，加快建设社会信用体系。完善统计机制，强化绩效考核，努力提高统计工作的科学化水平。加强国防后备力量建设。健全完善应急联动、灾情会商和信息共享机制等灾害应急管理体系建设，提高救灾应急处置能力，有效应对各类突发事件，为转型跨越发展创造良好的社会环境。

加强安全生产工作。严格落实各级政府领导“一岗双责”和部门安全监管责任，加大源头治理力度，坚决执行建设项目安全设施“三同时”和职业危害设施“三同时”制度，强力推进企业安全生产标准化建设。要进一步排查治理各类隐患，深入持久地抓好煤矿、非煤矿山、道路交通、危险化学品、特种设备、易燃易爆物品、森林防火和公共场所等各行业各领域安全生产专项整治。要严格安全目标责任考核，严格追究事故责任，坚决遏制重特大事故发生，把安全生产各项指标严格控制在省定指标以内，确保全市安全生产形势持续稳定。

（八）全面加强政府自身建设，在提升执行能力上取得新进展。

适应时代召唤、人民期待、社会需要，努力建设一个忧民所忧、乐民所乐的服务政府，务实高效、勤勉创新的责任政府，公正严明、规范有序的法治政府，风清气正、公开透明的廉洁政府。

坚持抓好学习，努力提升素质。坚持把学习作为一种政治责任和精神追求，时刻保持自觉学习的良好精神状态，努力做到学习工作化、工作学习化，通过学习激发工作活力、更新知识储备、创新思维方式，力求思想适应发展变化、知识适应时代要求、能力适应工作需要，切实提高驾驭复杂局面和解决实际问题的能力。大力提倡创造性、创新性、创意性工作，站在全市发展大局中谋划局部，运用改革创新的方法破解难题，使每一项工作都具有专业化品质，营造一种敢于突破创新、善于另辟蹊径的干事创业环境，力争使每一项事业都能在全省名列前茅。

坚持依法行政，创造良好环境。加快转变政府职能，注重提高服务效率和依法行政水平，努力建设服务型政府。树立程序意识，严格依照法定权限和程序履行职责、行使权力，切实做到严格、规范、公正、文明履职，努力增强政府的公信力。要改进执法方式，规范行政执法，严格落实执法责任制，营造良好的政务环境。大力推进政务公开，深入开展民主评议政风行风工作，完善政府新闻发布制度和信息公开制度，加强电子政务建设，让权力在阳光下运行。

坚持求真务实，狠抓工作落实。围绕工作重点，深入调查研究。各级领导既要当好战略家、设计师，又要当好战术家、工程师。要进一步强化责任意识，求真务实，敢于担当。要建立目标倒逼机制，使“请给我结果”成为每一个政府工作人员的座右铭。对重大事项、重要工作、重点项目，主要领导要紧紧抓在手上，走出会场，深入现场，开会开在现场，拍板拍在现场，落实落在现场。

坚持廉洁高效，严格监督考核。要进一步严格考核制度，抓好日常督查，强化各项重点工作落实的量化考核和监督检查，切实做到有布置、有检查、有考核、有奖惩。要坚持“三只看三不听”，即只看效果、只看数据、只看发展；不听过程、不听原因、不听解释，高标准高质量地推进各项工作。坚持以制度建设为主线，以权力监控为核心，建立健全惩治和预防腐败体系，从源头上防止腐败现象的发生。

在做好以上各项工作的同时，今年我们还要实施“方便晋城”十大工程：一是城乡社会保障一卡通工程；二是市县两级医院远程医疗会诊系统健康工程；三是新（改、扩）建15所标准化幼儿园的花蕾工程；四是全市普通高中教育免学费全覆盖希望工程；五是为农村义务教育寄宿制学校配备首批76辆校车的校安工程；六是硬化20条市区街巷、新建16座公厕的便利工程；七是完成主城区整体亮化工程；八是凤台街人行道改造提升畅通工程；九是新增供热面积150万平方米的温暖工程；十是取消陵沁一级公路西上庄收费站的撤卡工程。

各位代表！晋城正处于转型跨越发展的重要关头，这是我们的责任，这是我们的使命。我们要在中共晋城市委的坚强领导下，坚定信心，迎难而上，开拓进取，扎实工作，加快建设山西经济新的重要增长极和新兴产业重要支撑基地，奋力打造山西乃至中部地区最具发展活力，最有竞争实力，最富人文魅力，“方便、温馨、开放、现代”的宜居之城、活力之城、幸福之城，以优异成绩迎接党的十八大胜利召开！
